# Clinical and histopathological characteristics, diagnosis and treatment, and comorbidities of Bowen’s disease: a retrospective study

**DOI:** 10.3389/fmed.2023.1281540

**Published:** 2023-11-15

**Authors:** Cheng Zhou, Bin Jiang, Kaoyuan Zhang, Jingwen Wu, Cong Huang, Ning Xu, Tinglu Ye, Bancheng Chen, Bo Yu, Yanfen Zou, Changbing Shen

**Affiliations:** ^1^Department of Dermatology, Peking University Shenzhen Hospital, Shenzhen, Guangdong, China; ^2^Shenzhen Key Laboratory for Translational Medicine of Dermatology, Institute of Dermatology, Shenzhen Peking University - The Hong Kong University of Science and Technology Medical Center, Shenzhen, Guangdong, China

**Keywords:** Bowen’s disease, characteristic, diagnosis, treatment, comorbidity, retrospective study

## Abstract

**Background:**

Bowen’s disease (BD) is a slow-growing precancerous skin condition, often concurrent with other diseases, with a high misdiagnosis rate. Previous studies show that patients with BD in different populations have differentiated characteristics.

**Materials and methods:**

A retrospective study was conducted in a tertiary hospital in Shenzhen, China. Data about demographic information, diagnosis and treatment, clinical and pathological characteristics, and comorbidities of 50 patients with BD were collected and analyzed.

**Results:**

Clinical data of onset age and disease course of 43 patients with BD were available, the average onset age of male and female patients are 55.1 (standard deviation (SD) = 15.29) and 58.2 (SD = 15.59) years old, respectively; the average disease course of male and female patients are 25.3 (SD = 28.63) and 33.9 (SD = 49.65) months, respectively. The onset age (*p* = 0.52) and disease course (*p* = 0.49) between male and female patients are not significantly different. Interestingly, there is a negative correlation between onset age and disease course (r = −0.245, *p* = 0.11). The correct rate of clinical diagnosis is relatively low (54.00%); Some patients with BD are misdiagnosed as Bowenoid papulosis (10.00%), actinic keratosis (8.00%), basal cell carcinoma (8.00%), seborrheic keratosis (6.00%), and pigmented naevus (4.00%). Trunk and limbs are the most common distribution sites of BD lesions, and 94.00% patients with BD are treated with surgical resection; 66.00% patients with BD had comorbidities, including skin diseases (48.48%), cardiovascular diseases (39.39%), gastrointestinal diseases (30.30%), respiratory diseases (27.27%), and tumors (18.18%). The most commonly observed histopathological characteristics of BD are squamous-cell hyperplasia (86.00%), disordered maturation with atypical keratinocytes (74.00%), atypical mitoses (60.00%), hyperkeratosis with hypokeratosis (48.00%), dermal inflammatory cell infiltration (36.00%), and koilocytosis (22.00%).

**Conclusion:**

BD often occurs in middle-aged and elderly people and is easily misdiagnosed. The onset age and disease course of patients with BD are not significantly different between males and females, whereas there is a negative correlation between the onset age and disease course. BD is more likely to occur in trunk and limbs in the Chinese population, and most patients with BD are concurrent with comorbidities.

## 1 Introduction

Bowen’s disease (BD), also known as squamous cell carcinoma (SCC) *in situ*, is a slow-growing precancerous skin condition, and 3%–5% BD may progress to invasive tumors (1). The incidence of BD varies due to the difference in sun exposure in different latitudes and climates (2, 3). The etiology of BD is not entirely clear; it may be related to irradiation, carcinogens, human papillomavirus (HPV), and others (such as chronic injury, dermatoses, and heredity) (4–6). BD presents as a well-demarcated, asymptomatic, erythematous hyperkeratotic plaque with irregular margins that is more likely to occur on the exposed site in light-skinned people (5), which is difficult to differentiate from Bowenoid papulosis, actinic keratosis, and seborrheic keratosis. Clinically, patients with BD are concurrent with other diseases, but few studies have focused on the comorbidities of BD. In addition, there are differences in the characteristics of patients with BD in different populations. In order to improve the understanding of BD, we conducted a retrospective study in a tertiary hospital in China, the data about demographic information, clinical and pathological characteristics, diagnosis and treatment, and comorbidities of 50 patients with BD were collected and analyzed.

## 2 Materials and methods

### 2.1 Study design and participants

A retrospective study was conducted in a tertiary hospital (Peking University Shenzhen Hospital) in Shenzhen, China. In total, 50 patients with BD from outpatient or inpatient department were confirmed by histopathological examination from January 2016 to August 2023, including 26 males and 24 females. Demographic information, distribution of lesions, clinical diagnosis, onset age, disease course, treatment methods, histopathological characteristics, and comorbidities of BD were collected. The chronological age at the visit or admission time and the disease course were available in the majority of patients with BD; here, we obtained the onset age by chronological age minus the disease course. All patients with BD were called to recall whether there were some predisposing factors present at that time, including sun exposure, radiation, carcinogens, HPV infection, chronic injury, and genetic history. Meanwhile, all patients were called to inquire information about the recurrence of BD.

### 2.2 Division of the body sites and definition of comorbidities

In this study, the lesion distribution of BD in different parts of the body was studied. According to the conventional division method of the body, we divided the body into five parts: head and neck, upper limbs, trunk (including armpit and groin), lower limbs (including buttocks), and genital parts. The sun-exposed parts include ears, cheek, nose, forehead, opisthenar, and fingers. In addition, the comorbidities of patients with BD were collected for further analysis. Here, we defined “comorbidities” as those diseases that occurred before or during the course of BD; the diseases that occurred after the treatment time were not included.

### 2.3 Statistical analysis

Quantitative data were described by mean and standard deviation (SD), while qualitative data were described by number (N) and percentage (%). SPSS V22.0 (IBM Corp., Armonk, NY) and GraphPad Prism V9.0 (San Diego, CA) were used for statistical analysis. Q-Q plot was used to test whether the dataset followed a normal distribution or not. Continuous variables were evaluated by *t*-test or *t*-test with Welch’s correction according to the distribution of the dataset. The relationship between the onset age and disease course of patients with BD was calculated using Pearson’s correlation analysis. *p*-value <0.05 was considered statistically significant.

## 3 Results

### 3.1 Onset age, disease course, and skin lesion distribution of Bowen’s disease

**Figure 1:**
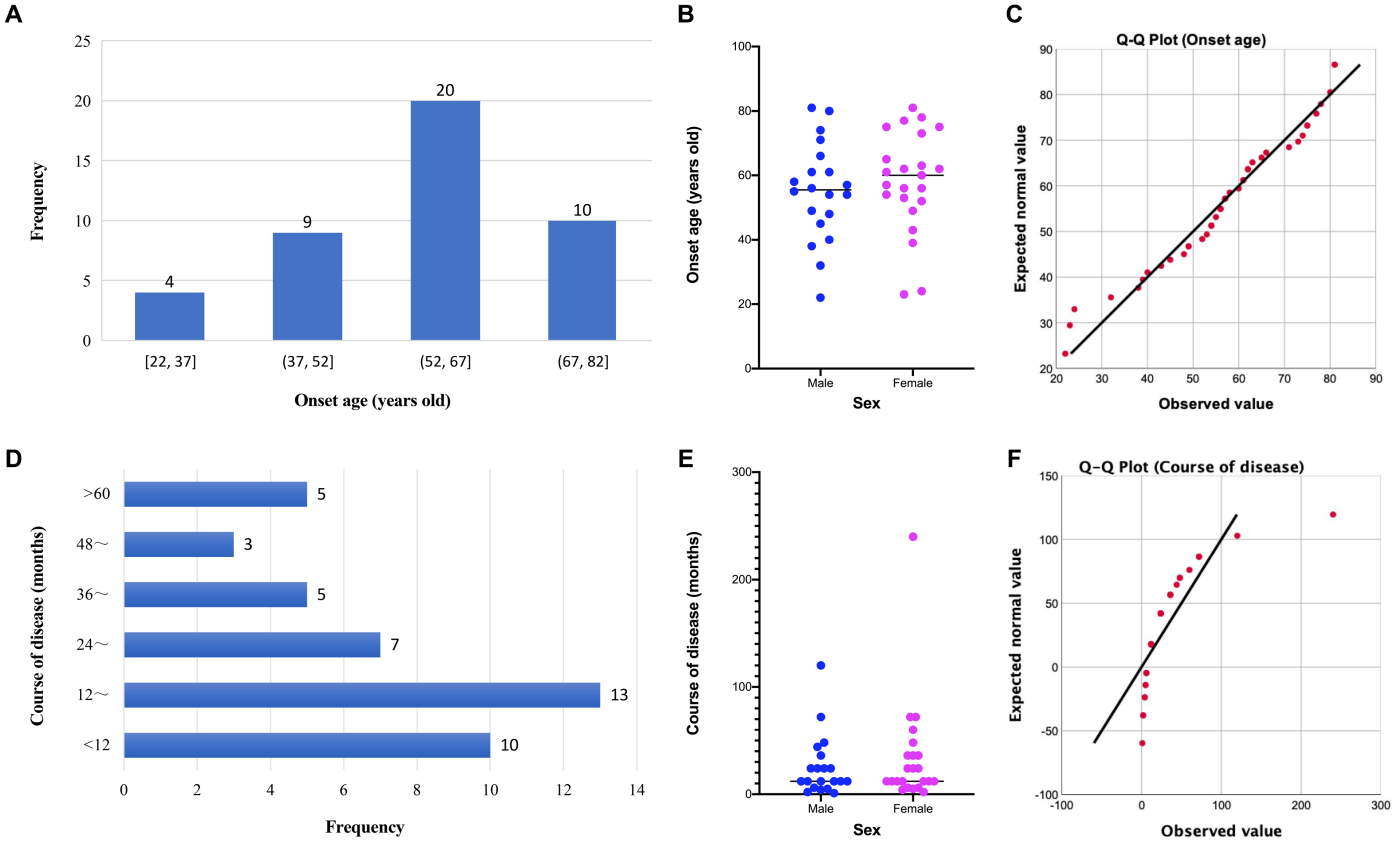
Distribution and comparison of onset age and disease course of patients with Bowen’s disease. **(A)** Distribution of onset age of patients with Bowen’s disease; **(B)** comparison of onset age of patients with Bowen’s disease between males and females; **(C)** Q-Q plot of onset age; **(D)** distribution of disease course of patients with Bowen’s disease; **(E)** comparison of disease course of patients with Bowen’s disease between males and females; **(F)** Q-Q plot of disease course.

Clinical data of 50 BD patients were collected. The patients’ chronological age ranged from 22 to 86 years old, with a mean age of 59.9 (SD = 14.66) years old. The onset age and disease course of 43 patients with BD were available for further analysis (Figure 1). The average onset age of 43 patients with BD is 56.7 (SD = 15.35) years old and that of male and female patients with BD are 55.1 (SD = 15.29) and 58.2 (SD = 15.59) years old, respectively. The disease course of 43 patients with BD ranges from 1 to 240 months, and the disease course of most patients is distributed in 12 to 24 months. The average disease course of 43 patients with BD is 29.9 (SD = 40.99) months and that of male and female patients are 25.3 (SD = 28.63) and 33.9 (SD = 49.65) months, respectively. The distribution of onset age is in accordance with normal distribution, and the distribution of disease course is approximately normal (Figure 1). The onset age of male and female patients is not significantly different by unpaired *t*-test (*F* = 1.04, *p* = 0.94; *t* = 0.65, *p* = 0.52). The difference in disease course between male and female patients is not significant by unpaired *t*-test with Welch’s correction (*F* = 3.01, *p* = 0.02; *t* = 0.70, *p* = 0.49). The correlation between onset age and disease course of patients with BD is negative (Pearson’s *r* = −0.245, *p* = 0.11). For the lesion distribution of BD (Table 1), trunk and limbs are the most commonly observed distribution sites; there are 26.00% patients with lesions in sun-exposed parts. In particular, 5 patients developed lesions on their fingers and 1 patient developed lesion on her feet.

**Table 1:** Lesion distribution of Bowen’s diseases.

Body parts	Subsites	Frequency	Frequency—total	Percentage	Cumulative frequency	Cumulative percentage
Head and neck	Ears^*^	3	6	12.00%	6	12.00%
	Cheek^*^	1				
	Nose^*^	1				
	Forehead^*^	1				
Upper limbs	Opisthenar^*^	2	10	20.00%	16	32.00%
	Upper arms	2				
	Forearm	1				
	Fingers^*^	5				
Trunk	Waist	6	16	32.00%	32	64.00%
	Back	6				
	Breast	2				
	Chest	1				
	Abdomen	1				
Genital	Vulva	4	7	14.00%	39	78.00%
	Penis	2				
	Foreskin	1				
Lower limbs	Lower legs	4	11	22.00%	50	100.00%
	Thigh	4				
	Pygal	1				
	Perianal region	1				
	Foot	1				

* Sun-exposed areas.

### 3.2 Clinical diagnosis and treatment methods of Bowen’s disease

The correct rate of clinical diagnosis is 54.00%. Some patients with BD are misdiagnosed as Bowenoid papulosis (10.00%), actinic keratosis (8.00%), basal cell carcinoma (8.00%), seborrheic keratosis (6.00%), and pigmented naevus (4.00%). One case each was diagnosed as verruca vulgaris, infectious granuloma, eczema, Paget’s disease, fibrous rash, glomus tumor, and SCC. For the treatment, 94.00% patients with BD received surgical treatment, and imiquimod cream was applied in 1 patient. 2 patients were treated with combined therapy, including surgery combined with photodynamic therapy (PDT) or liquid nitrogen cryotherapy. There were 30 patients with BD who gave feedback on information about recurrence, and 3 patients had a recurrence in the treatment area.

### 3.3 Predisposing factors and comorbidities of Bowen’s disease

In total, 30 patients with BD gave feedback on predisposing factors, and 5 patients with BD had trauma at the lesion locations. For the comorbidities of 50 patients with BD, 33 patients had comorbidities: 48.48% (16 of 33) patients were comorbid with skin diseases, including allergic dermatosis (*n* = 7), infectious dermatosis (*n* = 4), keloid (*n* = 2), hemangioma (*n* = 2), actinic keratosis (*n* = 2), and other skin diseases; 39.39% (10 of 33) patients were comorbid with cardiovascular diseases, of which hypertension was the most common comorbidity (*n* = 9); 30.30% (10 of 33) patients were comorbid with gastrointestinal disease, mainly chronic gastritis or proctitis (*n* = 7); 27.27% (9 of 33) patients were comorbid with respiratory diseases, including allergic rhinitis (*n* = 4), pharyngitis (*n* = 3), and bronchitis (*n* = 3); 18.18% (6 of 33) patients were comorbid with tumors. In addition, patients with BD were also comorbid with some other diseases, such as lumbar disk herniation, diabetes mellitus, benign prostatic hyperplasia or prostatitis, and hyperlipidemia; 21.21% patients with BD had 1 kind comorbidity, followed by 3 and 4 kinds of concomitant diseases with the same proportion (18.18%). It is worth noting that more than three-quarters of patients with BD had more than 2 kinds of comorbidities.

### 3.4 Histopathological characteristics of Bowen’s disease

The histopathological characteristics of 50 patients with BD were summarized, including squamous cell hyperplasia (86.00%), disordered maturation with atypical keratinocytes (74.00%), atypical mitoses (60.00%), hyperkeratosis with hypokeratosis (48.00%), dermal inflammatory cell infiltration (36.00%), and koilocytosis (22.00%). In addition, it also includes some other pathological characteristics, such as dyskeratosis, elastic fiber degeneration, pigment deposition, and scattered pigment incontinence.

## 4 Discussion

BD often occurs in middle-aged and elderly people; it is slightly more common in women (5). The sex ratio of male and female patients with BD was approximately 1:1 in our study. Of 50 patients with BD, the clinical data of onset age and disease course were available in 43 patients. The average onset age and average disease course of female patients with BD are higher than male patients, but the differences are not significant. We explored the correlation between onset age and disease course of patients with BD, result showing that these two with negative correlation, it means the elderly patients with BD had shorter disease course, it is possible that elderly people are more concerned about their own physical health.

BD is mostly involved in sun-exposed areas of the body and lesions often appear on the head, face, and limbs (7); more recent studies suggest BD has a predilection for the head and neck (8, 9). Trunk and limbs were the most common distribution, while lesions in the head and neck with the lowest proportion in our study, which is slightly different from previous findings (7–9). Interestingly, the lesions of 46.00% patients with BD located on non-sun-exposed sites, such as the trunk and genitals, suggest that we should pay more attention to rashes in non-sun-exposed sites. In particular, skin lesions of BD occurred on the fingers in 5 patients and on the feet in 1 patient. Previous studies indicated that HPV infection may be a potential risk factor for BD (10), multiple BD on the finger associated with HPV-34 (11) and HPV-16 (12), and BD on the dorsum of the foot associated with HPV-16 (13). Therefore, it is necessary to test HPV for BD occurring in the fingers and feet.

In clinical practice, the diagnosis of BD is usually made on the basis of clinical manifestations. Dermoscopy, as a non-invasive tool, is increasingly used in the clinical auxiliary diagnosis of BD (14). Ultrasound biomicroscopy (UBM) and high-frequency ultrasound (HFUS) also have potential as diagnostic tools for BD (15). Skin biopsy is often necessary to arrive at an accurate diagnosis of BD; all patients in our study underwent pathological examination. The correct rate of clinical diagnosis of BD before pathological examination is relatively low; BD is most easily misdiagnosed as Bowenoid papulosis, followed by actinic keratosis, basal cell carcinoma keratosis, seborrheic keratosis, and pigmented naevus. Interestingly, BD and seborrheic keratosis could be correctly identified by the deep learning model (area under the curve score > 0.97), the results of which should be confirmed by qualified histopathologists (16). Hopefully, more diagnostic methods with high accuracy will be developed in future.

The treatment methods for BD include topical therapies (5-fluorouracil (5-FU), imiquimod), cryotherapy (cryosurgery), curettage with cautery, PDT, standard surgical excision, Mohs micrographic surgery, laser (CO_2_ laser, non-ablative neodymium: YAG), radiotherapy, systemic treatments, and combination therapy (5). Some novel treatment approaches, such as pembrolizumab (a humanized monoclonal anti-PD-1 antibody for the treatment of melanoma and other malignancies) (17, 18) and thermotherapy (19), have also been tried for the treatment of BD. Previous studies reported that the complete clearance rate can reach 94.40% when the surgical margin is 5 mm (20). In this study, more than 90.00% patients with BD received surgical treatment, and two patients were treated with combined therapy, including surgery combined with PDT or cryotherapy. A previous study showed that PDT is a relatively effective treatment modality for BD, the overall clearance rate was 63.40% (21). An observational study compared different therapies: cryotherapy with the longest average treatment period, followed by imiquimod, PDT, and excision; surgical excision with the highest and PDT with the lowest therapeutic efficacy; imiquimod with recurrence rate was the highest (22). The efficacy of PDT in the treatment of BD varies among different studies, a meta-analysis shows that PDT treats BD with better efficacy, less recurrence, and better cosmetic outcomes than cryotherapy and 5-FU (23). Although a variety of methods have been applied to the treatment of BD, more therapeutic targets and methods need to be explored to enrich the treatment of BD in future.

More and more attention is being paid to the coexistence of diseases, but few studies focus on the comorbidity of BD. Previous studies reported that Merkel cell carcinoma (24, 25), cutaneous pseudolymphoma (26), breast cancer (27), and extramammary Paget’s disease (28), were concurrent with BD. In this study, 66.00% BD patients had comorbidities, mainly including skin diseases (48.48%), cardiovascular diseases (39.39%), gastrointestinal diseases (30.30%), respiratory diseases (27.27%), and tumors (18.18%). It is noticed that more than three-quarters of patients with BD had more than 2 kinds of comorbidities, and 1 patient had 14 kinds of comorbidities. Patients with so many kinds of comorbidities, which may be related to that most of the patients with BD are elderly people who have more underlying diseases. Therefore, it is necessary to pay attention to the comorbidities of patients with BD in diagnosis and treatment.

BD exhibits the histopathological features of full-thickness cell atypia, intact stratum basale, widened and elongated epidermal processes, and lymphocyte inflammatory cell infiltration around the superficial dermis vessels. In our study, the most common histopathological characteristic of BD is squamous cell hyperplasia, followed by disordered maturation with atypical keratinocytes, atypical mitoses, hyperkeratosis with hypokeratosis, dermal inflammatory cell infiltration, and koilocytosis. The summarization of these pathological characteristics is helpful for the diagnosis of BD in the Chinese population. Although there are some auxiliary diagnostic methods for the diagnosis of BD, histopathological examination is still necessary for the diagnosis of BD. The combination of fluorescence lifetime imaging microscopy (FLIM) and phasor approach (phasor-FLIM) is a screening tool for the differential diagnosis of BD, actinic keratosis, and basal cell carcinoma based on histopathological analysis (29). More diagnostic pathological characteristics need to be found in future.

## 5 Conclusions

BD often occurs in middle-aged and elderly people and is easily misdiagnosed clinically. The onset age and disease course of BD are not significantly different between males and females, whereas there is a negative correlation between the onset age and disease course of BD. BD is more likely to occur in the trunk and limbs in the Chinese population, and most BD patients are diagnosed with comorbidities. The associations between BD and its comorbidities should be further studied.

## Data availability statement

The raw data supporting the conclusions of this article will be made available by the authors, without undue reservation.

## Ethics statement

The studies involving humans were approved by Peking University Shenzhen Hospital. The studies were conducted in accordance with the local legislation and institutional requirements. The participants provided their written informed consent to participate in this study.

## Author contributions

CZ: Data curation, Formal analysis, Validation, Writing – original draft, Writing – review & editing. BJ: Data curation, Formal analysis, Validation, Writing – original draft, Writing – review & editing. KZ: Data curation, Writing – original draft, Writing – review & editing. JW: Data curation, Formal analysis, Validation, Writing – original draft, Writing – review & editing. CH: Data curation, Writing – original draft, Writing – review & editing. NX: Data curation, Writing - review & editing. TY: Resources, Writing – review & editing. BC: Resources, Supervision, Writing – review & editing. BY: Resources, Supervision, Writing – review & editing. YZ: Conceptualization, Resources, Supervision, Writing – review & editing. CS: Conceptualization, Data curation, Resources, Supervision, Writing – original draft, Writing - review & editing.
